# A *Lactobacilli* diet that confers MRSA resistance causes amino acid depletion and increased antioxidant levels in the *C. elegans* host

**DOI:** 10.3389/fmicb.2022.886206

**Published:** 2022-07-28

**Authors:** Katrine Vogt Møller, Hien Thi Thu Nguyen, Maria Grymer Metz Mørch, Marianne Overgaard Hesselager, Frans A. A. Mulder, Kurt Fuursted, Anders Olsen

**Affiliations:** ^1^Department of Chemistry and Bioscience, Aalborg University, Aalborg, Denmark; ^2^Department of Molecular Diagnostics, Aalborg University Hospital, Aalborg, Denmark; ^3^Interdisciplinary Nanoscience Center iNANO and Department of Chemistry, Aarhus University, Aarhus, Denmark; ^4^Statens Serum Institute, Copenhagen, Denmark

**Keywords:** *Caenorhabditis elegans*, *Lactobacillus*, MRSA, NMR, metabolomics, amino acids, probiotic bacteria

## Abstract

Probiotic bacteria are increasingly popular as dietary supplements and have the potential as alternatives to traditional antibiotics. We have recently shown that pretreatment with *Lactobacillus* spp. Lb21 increases the life span of *C. elegans* and results in resistance toward pathogenic methicillin-resistant *Staphylococcus aureus* (MRSA). The Lb21-mediated MRSA resistance is dependent on the DBL-1 ligand of the TGF-β signaling pathway. However, the underlying changes at the metabolite level are not understood which limits the application of probiotic bacteria as timely alternatives to traditional antibiotics. In this study, we have performed untargeted nuclear magnetic resonance-based metabolic profiling. We report the metabolomes of *Lactobacillus* spp. Lb21 and control *E. coli* OP50 bacteria as well as the nematode-host metabolomes after feeding with these diets. We identify 48 metabolites in the bacteria samples and 51 metabolites in the nematode samples and 63 across all samples. Compared to the control diet, the *Lactobacilli* pretreatment significantly alters the metabolic profile of the worms. Through sparse Partial Least Squares discriminant analyses, we identify the 20 most important metabolites distinguishing probiotics from the regular OP50 food and worms fed the two different bacterial diets, respectively. Among the changed metabolites, we find lower levels of essential amino acids as well as increased levels of the antioxidants, ascorbate, and glutathione. Since the probiotic diet offers significant protection against MRSA, these metabolites could provide novel ways of combatting MRSA infections.

## Introduction

Since the discovery of antibiotic substances, bacteria have shown an incredible ability to overcome the obstacle by developing resistance. In fact, according to the most recent antibiotic resistance threat report, more than 35,000 people die each year in the United States due to infections by multidrug-resistant bacteria (MDR) (CDC, 2019). MDR bacteria carry several resistance genes and they are evolving in line with the extensive use of antibiotics. Methicillin-resistant *Staphylococcus aureus* (MRSA) is an opportunistic pathogen most often residing in the upper respiratory passages without causing symptoms to the host (Lee et al., 2018). MRSA represents a risk to immune compromised individuals, potentially leading to severe illness and mortality, and carriers transmitting the bacteria without knowing that it is a huge clinical threat (Turner et al., 2019). To avoid antibiotic resistance and ameliorate the increasing threat from MDR bacteria, like MRSA, we require alternatives to traditional antibiotics.

Probiotics might offer such an alternative (Markowiak and Slizewska, 2017). They are defined as live microorganisms that confer a beneficial health effect on the host, when administered in adequate amounts (Hill et al., 2014). The use of probiotics specifically against MRSA is not new and has been evaluated in many different model systems and organisms including humans. It is clear that many different probiotic actions can offer protection against MRSA. These include but are not limited to antibacterial, anti-biofilm, anti-virulence, and antidrug resistance, improved intestinal barrier function, and host immune system stimulation (Sikorska and Smoragiewicz, 2013; Johansson et al., 2016; Nataraj and Mallappa, 2021). In addition to these protective mechanisms, probiotic bacteria can also outcompete MRSA by competition of binding sites or displacement. Interestingly, it has been reported that the consumption of probiotic *Bacillus* bacteria abolishes *S. aureus* nasal and intestinal colonization in a rural Thai population (Piewngam et al., 2018). The protective effect is a consequence of *Bacillus* produced fengycins, lipopeptides capable of inhibiting *S. aureus* quorum sensing (Piewngam et al., 2018). Another study in humans found that the daily oral administration of a *Lactobacillus rhamnosus* reduced odds of carriage of *S. aureus* in the GI tract (Eggers et al., 2018). This finding is consistent with a smaller study showing that oral and nasal spray administration of a probiotic *Lactobacilli* mixture displaced MRSA in long-term carriers (Roos et al., 2011).

The underlying molecular mechanisms are not always well-understood and to fully exploit the potential of probiotics as an alternative to traditional antibiotics, a deeper understanding is needed. Metabolomics, the profiling of metabolites, is one way to investigate the consequence of dietary supplementation of probiotics at the most downstream level of the cellular machinery. Metabolomics can thus provide insights to causal biological pathways and lead to identification of novel biomarkers (Johnson et al., 2016).

The rather complex interplay between humans and probiotic, commensal, and pathogenic bacteria makes simple model organisms, such as the small nematode *Caenorhabditis elegans*, attractive alternatives to study host-microorganism interactions. We recently identified *Lactobacillus* spp. Lb21 (a mix of *Lactiplantibacillus plantarum* and *Levilactobacillus brevis*) in a screen aimed at finding novel potentially probiotic bacteria (Mørch et al., 2021). The *Lactobacillus* spp. Lb21 (from now referred to as Lb21) was shown to extend the life span of *C. elegans* and probiotic pretreatment significantly increased the MRSA resistance. Epistasis analysis showed that the protective effect of Lb21 is mediated through the TGF-β related ligand DBL-1. Furthermore, proteomic analysis identified 308 regulated proteins in worms fed Lb21 vs. worms fed standard *E. coli* food (OP50). Interestingly, several studies have observed increased life span and improved resistance toward infections with other pathogenic bacteria when feeding *C. elegans* probiotic bacteria (Ikeda et al., 2007; Kim and Mylonakis, 2012; Park et al., 2014, 2018; Nakagawa et al., 2016; Christensen et al., 2017). Thus, their potential is not limited to MRSA.

Only a few studies have looked at the metabolome of *C. elegans* in relation to different food sources. Two studies compared the metabolic profile of *C. elegans* fed two different *E. coli* strains, namely, OP50 and HT115 (Reinke et al., 2010; Gao et al., 2017). These strains are typically used as standard food sources for the worm in regular and RNAi experiments, respectively. Interestingly, the identified metabolic profiles were different, and the changes were distinct enough to group the strains according to diet. The effect of a probiotic *Bifidobacterium animalis* subsp. *lactis* food source on the *C. elegans* metabolome has also been examined (Martorell et al., 2016). The strain reduces fat content, and the metabolic analysis suggests that it modulates both antioxidant responses and lipid metabolism of the worms.

In this study, we investigate how the metabolome of probiotic Lb21 differs from the regular OP50 bacteria. Furthermore, we characterize the changes in the host metabolome of *C. elegans* following a probiotic Lb21 diet compared to an OP50 diet. We find that worms being fed Lb21 have reduced levels of essential amino acids and contain a higher level of the antioxidant glutathione.

## Materials and methods

### Nematode and bacterial strains

The *C. elegans* strain used in this study, NL2099 *rrf-3 (pk1426)*, was purchased from the *Caenorhabditis* Genetics Center (CGC) at the University of Minnesota. This mutant is sterile when cultured at 25°C and used to prevent offspring cross-contaminating and prevent bagging.

The following three different bacterial strains were used in this study: the standard *C. elegans* laboratory food strain *Escherichia coli* OP50 (Brenner, 1974); Lb21, a mix of two *Lactobacillus* strains [*L. plantarum* (LX0811-1) and *L. brevis* (LX0811-2)] provided by DuPont Nutrition Biosciences ApS, now IFF: International Flavors & Fragrances (Brabrand Denmark); and a community-acquired isolate MRSA 43484 provided by from Statens Serum Institut, Copenhagen, Denmark.

### Culturing conditions

Bacteria were cultured as previously described (Mørch et al., 2021). Briefly, OP50 and MRSA 43484 stocks were kept on Luria-Bertani (LB) and Tryptic Soy (TS) agar plates (Sigma), respectively, grown ON at 37°C and stored at 5°C. Lb21 was made from stock and stored as aliquots at −80°C in 25% glycerol.

Overnight (ON) bacterial cultures were prepared by inoculation of single colonies into 10 ml of LB or 10 ml of TS broth for OP50 and MRSA 43484, respectively. Lb21 was prepared by inoculation of 75 μl of aliquot into 10 ml De Man, Rogosa, and Sharpe (MRS) broth (Sigma). All cultures were grown under aerobic conditions for 18 h at 37°C in 13 ml of bacterial culture tubes under continuous shaking at 190 rpm.

Large bacterial cultures of OP50 and Lb21 were made by inoculating 1L (LB/MRS) broth with 2 ml of ON culture and grown under aerobic conditions for 18 h at 37°C under continuous shaking at 100 rpm. Cultures were concentrated 4:1 and spotted on large nematode growth media (NGM) plates (Brenner, 1974) to be used for both lawn samples and worm samples and for pathogen life span analysis. Bacterial lawns grew on the plates at room temperature for 6 days before being used for *ad libitum* feeding or harvest.

To generate a large population, worms were cultured on 9 cm NGM plates with concentrated OP50 (40X) at 20°C. Gravid adults were washed off with S-basal (10 M NaCl, 0.05 M KPO_4_ in H_2_O) and the supernatant was removed before the worm pellet was treated with an alkaline hypochlorite solution (0.25 M KOH, ~2% sodium hypochlorite (10-15%, Acros Organics) to harvest the eggs and get a synchronous population. A total of 500,000 eggs were divided between 225 plates with UV-irradiated OP50 (OP50-UV). OP50-UV plates were irradiated for 30 min on the previous day, and on the day of hypo-treatment in a UV cross-linker (UVP). This was to prevent residual bacteria from dividing when worms were later transferred to Lb21. The worms were allowed to develop at 25°C for 3 days to get sterile adults. Three-day-old worms were washed off from OP50-UV plates, washed three times in S-basal, and transferred to either OP50 or Lb21. The plates were incubated at 25°C for 48 h before a part of the population was shifted to MRSA 43484 plates for life span analysis and the rest was used for metabolite extraction.

### Pathogen life span assay

Worms that have been pretreated with OP50 or Lb21 for 48 h were transferred to MRSA plates on day 5. Notably, 50 worms per plate and five plates per group were used. Death events were scored every other day. Worms were incubated at 25°C. The GraphPad PRISM software was used to plot data and perform log-rank tests and the Gehan-Breslow-Wilcoxon tests.

### Samples for metabolic studies

A T-shaped spreader was used to scrape off the lawns and transfer the bacteria to ice-cold isotonic saline (0.9%) solution (ISS). A total of 17 large NGM plates were used for each bacterial lawn sample and three samples of each group were made. Tubes were centrifuged for 10 min at 4,000 rpm at 4°C, supernatants were discarded, and pellets were washed two additional times in ice-cold ISS. Pellets were snap-frozen in liquid nitrogen and stored at −80°C. Before extraction, OD_600_ was measured, and all samples were diluted to the same OD.

Five-day-old worms were sampled for NMR. Approximately, 20 large (9 cm) plates were pooled for each sample, containing around 11,500 worms, by washing them off with S-basal. The worms were left to settle and washed three additional times before pellets were snap-frozen in liquid nitrogen and stored at −80°C. Three samples of each group were made.

### Extraction of metabolites

Bacteria: 2 ml of 80% methanol was added to each pellet, and then subjected to four freeze-thaw cycles in liquid nitrogen and hot water to open the cells. The samples were vortexed after being thawed. Finally, the tubes were centrifuged for 10 min at 4,000 rpm, 4°C, and the extracts were dried in a speedVac (Thermo Scientific) and then kept at −80°C until analysis.

Worms: Metabolites were extracted following the method described by Geier et al. (2011) using 80% methanol in water and bead-beating. Briefly, pellets in ice-cold 80% methanol were bead-beaten in a Precellys 24 tissue homogenizer (Bertin-instruments) using glass beads of various sizes (100 mg of each; 0.1 mm, 0.45 mm, and 1.0 mm) to break open the cells of the worms. The extracts were dried as described for bacteria.

### NMR spectroscopy

Dried pellets were dissolved in 550 μl of NMR buffer [0.1 M phosphate buffer, pH 7.0, 10% D_2_O and 0.25 mM 4,4-dimethyl-4-silapentane-1-sulfonic acid (DSS)]. A volume of 500 μl was transferred to NMR tubes suited for use with Bruker SampleJet. ^1^H spectra were acquired at 295 K using a 1D NOESY pulse sequence with water pre-saturation on a Bruker 500 MHz NMR spectrometer with a room temperature TXI probe head, collecting 8 scans per sample. A relaxation delay of 2 s and a mixing time of 50 ms were used, collecting 32 K data points over a spectral width of 16 ppm, at an acquisition time of 2.04 s. 2D NMR ^1^H-^1^H COSY spectra were acquired to annotate peaks in overlapping regions. Here, 64 scans were collected, using a relaxation delay of 1.5 s, acquiring 16 K data points over a spectral width of 13 ppm, at an acquisition time of 0.63 s.

### NMR data processing and analysis

For metabolite identification, two-dimensional (2D) experiments were performed. Information obtained from these spectra was used to find matching metabolites with the Human Metabolome Database (Wishart et al., 2007). Further metabolite assignments were performed using the Chenomx NMR Suite 8.1 professional software (Chenomx Inc., Edmonton, AB, Canada). NMR spectra were aligned and normalized to the DSS spectral area for calculating the concentration of each metabolite. Regions containing residual water (δ 4.825–4.725 ppm) were excluded from the dataset to avoid spectral interference.

### Statistical analysis

In this study, the modified MetaboAnalystR 2.0 R package (https://github.com/biocyberman/MetaboAnalystR/tree/dev) was applied for all univariate and multivariate statistical analyses.

An overview of the data and a rough ranking of potential important features were first obtained using univariate analysis. Univariate analysis examines each variable separately without considering the effect of multiple comparisons. The results provided by all univariate methods were further examined using a more sophisticated analysis tool.

We performed multivariate statistical analysis with unsupervised Principal Components Analysis (PCA) and sparse Partial Least Squares discriminant analysis (sPLS-DA). These two methods are the most popular unsupervised and supervised methods, respectively, for the multivariate statistical analysis of metabolomics data where the NMR data are projected into a reduced dimensional space for easier interpretation and visualization (Ebbels et al., 2011). In PCA, the model describes the space corresponding to the highest variance of the data, while in sPLS, the space corresponds to that with the highest covariance between the NMR data and the response variable (Puchades-Carrasco et al., 2016). sPLS has been developed to perform simultaneous variable selection in both X and Y data sets, by including least absolute shrinkage and selection operator (LASSO) ℓ1 penalizations in PLS on each pair of loading (Lê Cao et al., 2008). sPLS was shown to be effective and biologically meaningful, and it provides a valuable variable selection tool for high dimensional data sets compared to PLS (Lê Cao et al., 2008, 2011). First, the metabolite profile analysis was performed using unsupervised PCA, which displayed the internal structure of datasets in an unbiased way and decreases the dimensionality of data. Then, the sPLS-DA model was employed to identify the metabolites contributing to discrimination between groups. The loading plot indicated the contribution of each metabolite to the model. Notably, 5-fold cross-validation together with the receiver operating characteristic curve (ROC) was chosen for the evaluation of classification performance of the sPLS-DA model. The heat map of significantly different features was generated to identify clustering metabolites.

## Results

### Metabolic profiling of Lb21

**Figure 1:**
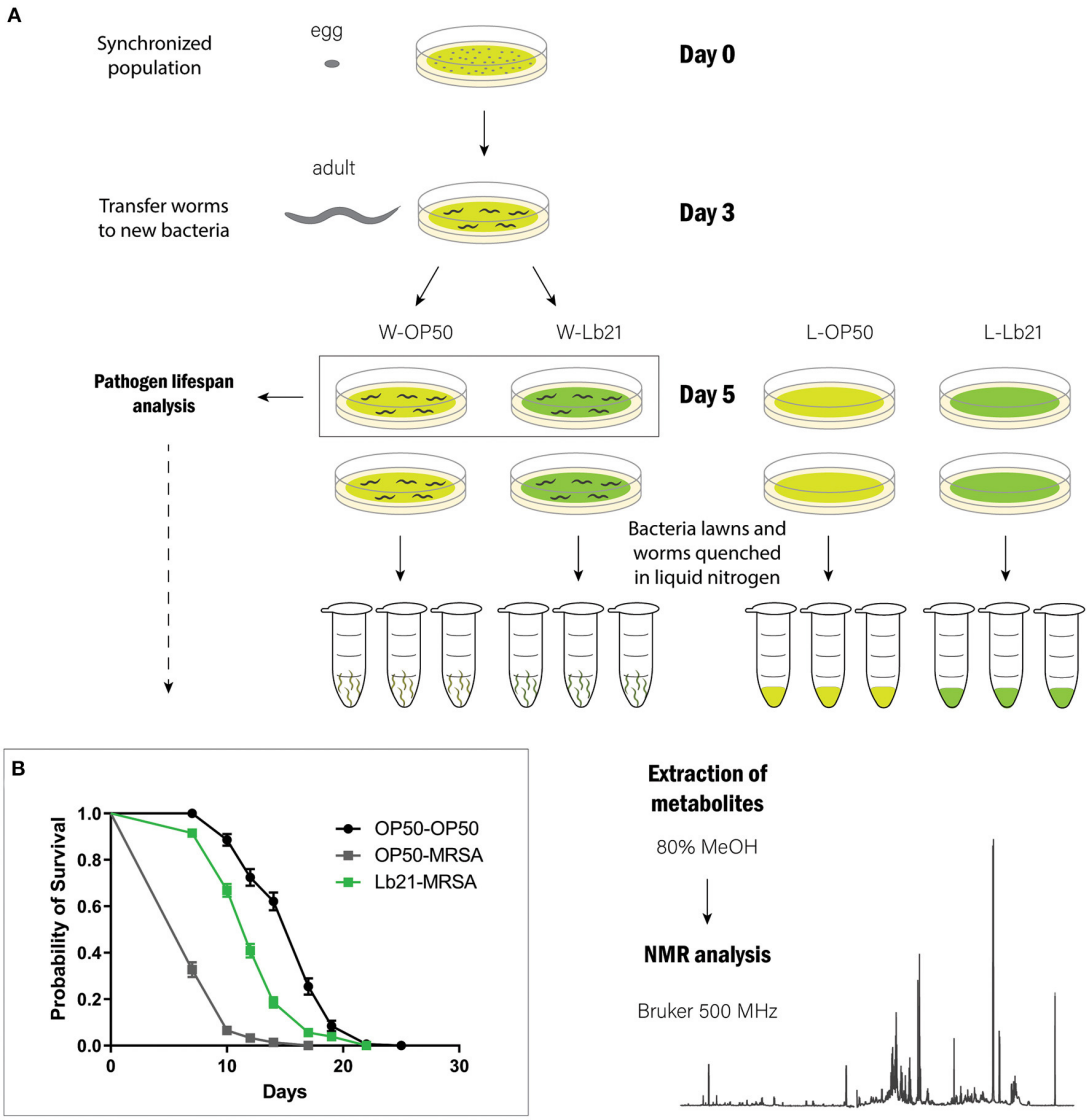
Experimental design: **(A)** A synchronized population of rrf-3 worms was obtained by bleaching gravid adults. ~500.000 eggs were distributed onto 225 large agar plates spotted with *E. coli* OP50 (UV irradiated for 30 min) on day 0. On day 3, adult worms were transferred to *E. coli* OP50 or Lb21. On day 5, a subset of the worms was transferred to plates spotted with MRSA for pathogen resistance analysis. The remaining worms and bacterial lawns were prepared in triplicate to extract metabolites. **(B)** MRSA resistance analysis. Worms pretreated with Lb21 lived significantly longer (*p* < 0.0001) than worms fed the standard OP50 bacteria when challenged with MRSA. Mean life span (days) of OP50-MRSA worms was 8.2 ± 2.0 (*n* = 214) compared to 12.7 ± 3.4 (*n* = 284) following Lb21 pretreatment.

To gain further insight into the underlying probiotic mechanism of Lb21, we decided to characterize the metabolic profiles of both standard OP50 and probiotic bacteria, as well as metabolic profiles from worms fed the two different food sources. To obtain metabolic profiles of both bacteria and worms mimicking the experimental setup showing probiotic effects, the bacteria were spotted onto agar plates and scraped off and worms were cultured on large plates and not in liquid culture (Figure 1A).

To verify a positive effect of Lb21 under these mass culture conditions, a pathogen resistance analysis was performed simultaneously. As expected, significant resistance toward MRSA infection was observed following pretreatment with Lb21 (Figure 1B). This is consistent with our earlier observations using small NGM plates (Mørch et al., 2021).

**Figure 2:**
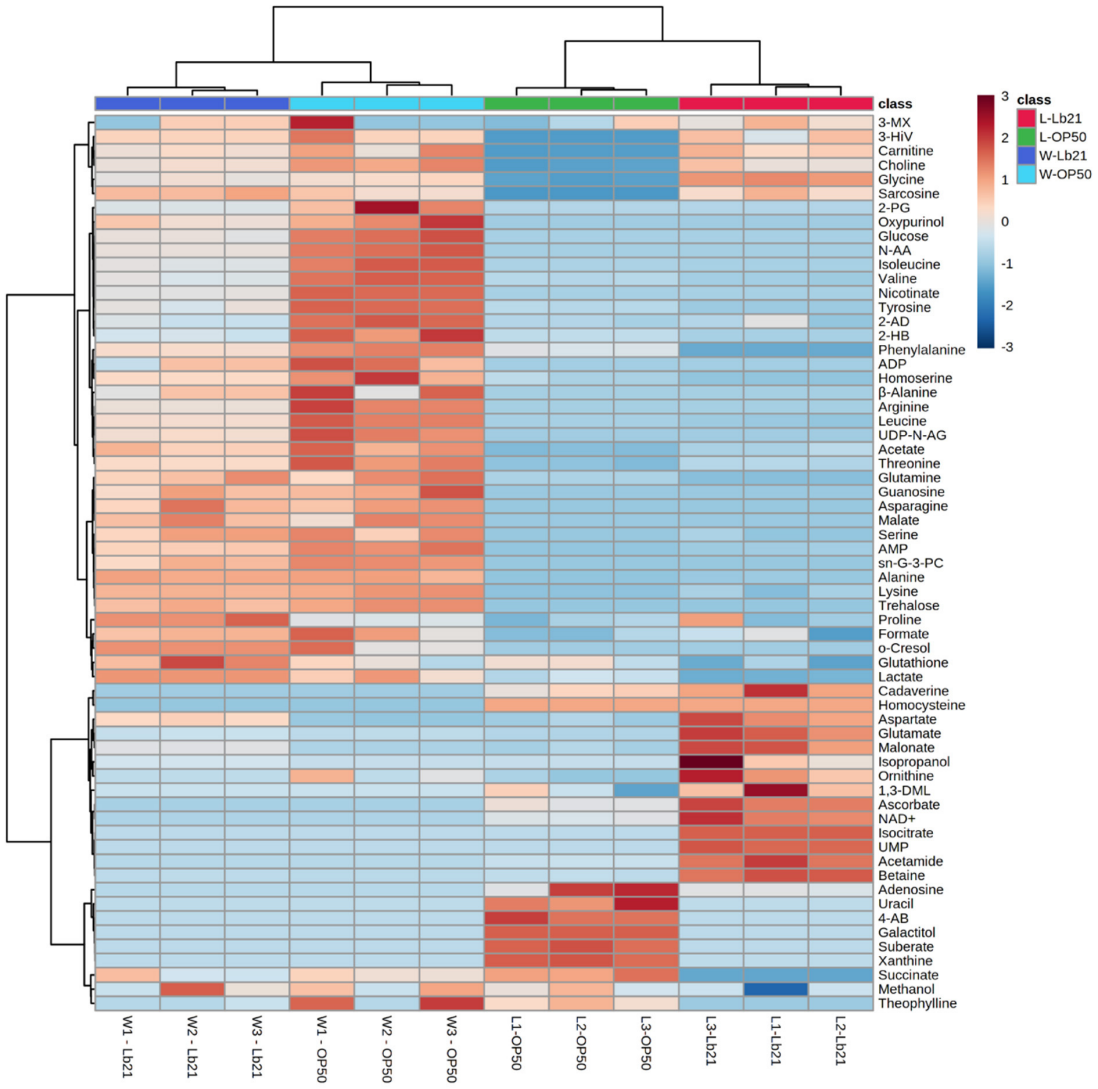
Host metabolic profiles do not simply reflect the metabolic profile of the bacterial diets: Heat map representing the hierarchical clustering of all the assigned metabolites in four different sample groups, each made in triplicate. Bacterial lawn samples are shown to the right (L-OP50 and L-Lb21) and worms samples to the left (W-OP50 and W-Lb21). Each row shows data for a specific metabolite, and each column shows data for bacteria and worms. Red and blue denote relatively high and low levels, respectively, compared to the average level.

Endogenous metabolites of both bacteria and worm samples were extracted and measured using ^1^H NMR spectroscopy. A total of 63 metabolites were assigned, with 48 metabolites for the bacterial samples and 51 for the worm samples. A heat map with hierarchical clustering of the total assigned metabolites that differed significantly between groups demonstrates the ability of the metabolomics approach to differentiate between different groups since the four groups clustered separately (Figure 2).

**Figure 3:**
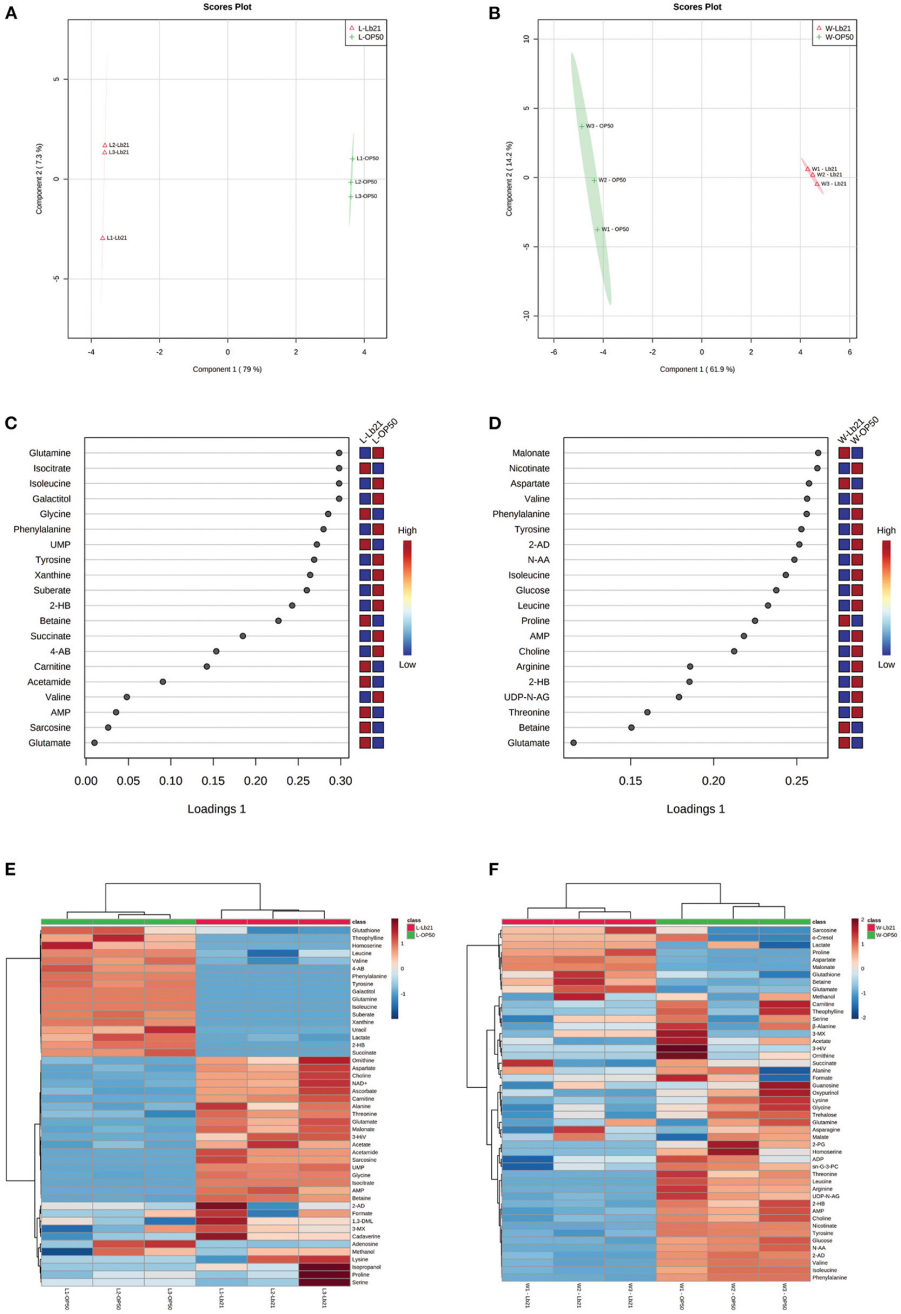
sPLS-DA and hierarchical clustering analysis: sPLS-DA score plot and loading plot from bacterial lawn samples and from worms fed the two different bacteria. **(A)** sPLS-DA score plot from *E. coli* strain OP50 (L-OP50, green) vs. *Lactobacillus* spp. Lb21 (L-Lb21, pink). **(B)** sPLS-DA score plot from worms fed different bacteria, *E. coli* strain OP50 (W-OP50, green) vs. Lb21 (W-Lb21, pink). **(C)** Loading plot from the bacterial strains, L-OP50 vs. L-Lb21 **(D)** Loading plot from worms fed OP50 or Lb21 (W-OP50 vs. W-Lb21). Hierarchical clustering analysis highlighted significant differences between bacterial lawn groups, L-OP50 vs. L-Lb21 **(E)**; and worms fed the two different bacteria groups, W-OP50 vs. W-Lb21 **(F)**.

The endogenous metabolic variances from bacterial lawn samples (L-OP50 and L-Lb21) and from worms fed the two different bacteria (W-OP50 and W-Lb21) were analyzed by the sPLS-DA method. The sPLS-DA models were able to separate the L-OP50 group from the L-Lb21 group (Figure 3A), and the W-OP50 group from the W-Lb21 group (Figure 3B). The sPLS-DA explained 86.3% and 76.1% of variance with the first two components in the samples collected for the two models, respectively. The sPLS-DA model for both shows separation with an overall cross validated accuracy of 100% (data not shown). The loading plots display the top twenty metabolites responsible for the differentiation of the groups in a ranked order (Figures 3C,D). Hierarchical clustering analysis highlighted the significant differences between bacterial lawn groups, L-OP50 vs. L-Lb21 (Figure 3E) and worms fed the two different bacteria, W-OP50 vs. W-Lb21 (Figure 3F).

Several amino acids (AAs) were identified as important factors for the separation of OP50 and Lb21 bacterial lawns and for distinguishing the worms fed the two bacterial diets (Figures 3C,D). Five of these AAs were present in all four groups (i.e., isoleucine, valine, phenylalanine, glutamate, and tyrosine). The levels of the three essential AAs, namely, isoleucine, valine, and phenylalanine, and the non-essential tyrosine were significantly lower in Lb21 and worms fed Lb21 compared to OP50 and worms fed OP50 (Tables 1, 2). In contrast, the non-essential AA glutamate was significantly higher.

**Table 1:** Important features selected by *t*-tests for separation of L-OP50 and L-Lb21.

	**Compounds**	**t-stat**	***p*** **-value**	−**log10(p)**	**FDR**	**Description**
1	Galactitol	−418	1.9653e-10	9.7066	9.2369e-09	Energy, alcohol form of galactose.
2	Isocitrate	131.2	2.0242e-08	7.6938	4.7568e-07	Energy, intermediate in TCA cycle.
3	Isoleucine	−62	4.0535e-07	6.3922	6.3505e-06	Amino acid (essential).
4	Phenylalanine	−48.204	1.1081e-06	5.9554	1.2071e-05	Amino acid (essential).
5	Glycine	46.457	1.2842e-06	5.8914	1.2071e-07	Amino acid.
6	UMP	40.286	2.2686e-06	5.6442	1.7771e-05	Nucleotide.
7	Tyrosine	−37.812	2.9216e-06	5.5344	1.9616e-05	Amino acid.
8	Xanthine	−34.842	4.0493e-06	5.3926	2.379e-05	Purine base. Degradation of purines.
9	Suberate	−33.272	4.8664e-06	5.3128	2.5414e-05	Organic acid.
10	Glutamine	−30.368	7.0037e-06	5.1547	3.2918e-05	Amino acid.
11	2-Hydroxybutyrate	−22.357	2.3698e-05	4.6253	0.00010126	Ketone body.
12	Betaine	19.648	3.9573e-05	4.4026	0.00015499	Osmoprotectant. Methyl donor to form methionine from homocysteine. Precursor of glycine, serine, and threonine.
13	4-AB (GABA)	−16.857	7.2593e-05	4.1391	0.00026245	Confers resistance toward acidic pH.
14	Succinate	−15.565	9.9465e-05	4.0023	0.00033392	Energy, intermediate in TCA cycle.
15	Carnitine	13.243	0.00018786	3.7262	0.00058863	Osmoprotectant.
16	AMP	12.108	0.0002669	3.5736	0.00076424	Nucleotide.
17	Sarcosine	12	0.00027643	3.5584	0.00076424	Amino acid. N-methyl derivative of glycine.
18	Acetamide	11.442	0.00033292	3.4777	0.00086929	Compound used in the production of plastics.
19	Valine	−10.401	0.00048261	3.3164	0.0011938	Amino acid (essential).
20	Glutamate	9.6687	0.00064022	3.1937	0.0015045	Amino acid.
21	Threonine	9.4997	0.00068531	3.1641	0.0015338	Amino acid (essential).
22	Lactate	−8.8206	0.00091165	3.0402	0.0019476	Energy.
23	Malonate	8.5982	0.0010054	2.9977	0.0020545	Competitive inhibitor of succinate dehydrogenase. Used in fatty acid or β-alanine biosynthesis.
24	Choline	8.2549	0.0011748	2.93	0.0023007	Oxidized to betaine. Cation present in phospholipids. Precursor of neurotransmitter acetylcholine.
25	Ascorbate	8.1536	0.0012314	2.9096	0.0023151	Antioxidant.
26	Aspartate	7.8227	0.0014415	2.8412	0.0026059	Amino acid.
27	Uracil	−7.3321	0.0018417	2.7348	0.0031305	Nucleobase.
28	3-HiV	7.3077	0.001865	2.7293	0.0031305	Organic acid.
29	Theophylline	−6.9322	0.0022735	2.6433	0.0036847	A xanthine alkaloid.
30	NAD+	6.6574	0.0026441	2.5777	0.0041425	Coenzyme.
31	Acetate	6.3614	0.0031302	2.5044	0.0047458	Building block for biosynthesis, such as fatty acids.
32	Homoserine	−6.1418	0.0035635	2.4481	0.0052338	α-amino acid.
33	Alanine	4.9031	0.0080258	2.0955	0.011431	Amino acid.
34	Leucine	−4.818	0.0085357	2.0688	0.011799	Amino acid (essential).
35	Ornithine	4.3704	0.011964	1.9221	0.016066	Non-proteinogenic amino acid. Plays a role in the urea cycle.
36	Glutathione	−3.3966	0.027362	1.5628	0.035723	Antioxidant.

**Table 2:** Important features selected by *t*-tests for separation of W-OP50 and W-Lb21.

	**Compounds**	**t-stat**	***p*** **-value**	−**log10(p)**	**FDR**	**Description**
1	Malonate	72.458	2.174e-07	6.6627	1.0435e-05	Competitive inhibitor of succinate dehydrogenase. Used in fatty acid or β-alanine biosynthesis.
2	Nicotinate	−56	6.088e-07	6.2155	1.4611e-05	Precursors for coenzymes, NAD^+^ and NADP^+^.
3	Aspartate	24.922	1.5387e-05	4.8128	0.00022291	Amino acid.
4	Valine	−22.858	2.1702e-05	4.6635	0.00022291	Amino acid (essential).
5	Phenylalanine	−22.472	2.3219e-05	4.6342	0.00022291	Amino acid (essential).
6	Tyrosine	−18.882	4.6337e-05	4.3341	0.00037069	Amino acid.
7	2-Aminoadipate	−17.898	5.7278e-05	4.242	0.00039276	Intermediate in lysine biosynthesis/degradation.
8	N-Acetylaspartate	−15.99	8.9428e-05	4.0485	0.00053657	Derivative of aspartic acid.
9	Isoleucine	−13.765	0.00016139	3.7921	0.00086074	Amino acid (essential).
10	Glucose	−12.127	0.00026527	3.5763	0.0012733	Energy.
11	Leucine	−11.088	0.00037625	3.4245	0.0016418	Amino acid (essential).
12	Proline	9.877	0.00058957	3.2295	0.0023583	Amino acid.
13	AMP	−9.0994	0.00080896	3.0921	0.0029869	Nucleotide, energy.
14	Choline	−8.5413	0.0010313	2.9866	0.0035358	Cation present in phospholipids. Oxidized to betaine. Precursor of neurotransmitter acetylcholine.
15	Arginine	−6.8636	0.0023596	2.6272	0.0071505	Amino acid (essential).
16	2-Hydroxybutyrate	−6.8452	0.0023835	2.6228	0.0071505	Ketone body.
17	UDP-N-AG	−3.9973	0.016166	1.7914	0.0078547	Nucleotide sugar and coenzyme.
18	Threonine	−5.8883	0.0041589	2.381	0.011091	Amino acid (essential).
19	Betaine	5.6095	0.0049615	2.3044	0.012534	Methyl donor to form methionine from homocysteine. Precursor of glycine, serine, and threonine.
20	Glutamate	4.8347	0.0084325	2.074	0.020238	Amino acid.
21	Sn-Glycero-3-PC	−3.4429	0.026225	1.5813	0.059943	Nootropic phospholipid, a precursor to choline biosynthesis.
22	2-Phosphoglycerate	−3.166	0.033989	1.4687	0.070993	Substrate in glycolysis.
23	Lysine				0.070993	Amino acid (essential).
24	Trehalose	−3.0823	0.036851	1.4336	0.070993	Sugar.
25	Glutathione				0.070993	Antioxidant.
26	Homoserine	−2.9571	0.041673	1.3801	0.070993	α-amino acid.
27	Sarcosine	2.9492	0.042001	1.3767	0.070993	Amino acid. N-methyl derivative of glycine.
28	Oxypurinol	−2.9446	0.042196	1.3747	0.070993	Purine derivative.
29	Glycine	−2.9282	0.042892	1.3676	0.070993	Amino acid.

A shift was observed for threonine, an essential AA, which was significantly higher in L-Lb21 compared to L-OP50 (Table 1), although the worms fed Lb21 had significantly lower levels of threonine compared to worms fed OP50 (Table 2).

Glutamine was only identified as an important factor for separating L-Lb21 from L-OP50 and the concentration was significantly lower in L-Lb21 (Figure 3C and Table 1). Worms fed Lb21 compared to worms fed standard OP50 food had significantly increased levels of aspartate and proline, and a significantly lower concentration of arginine and leucine (Figure 3D and Table 2).

In addition to these AAs, other metabolites were identified as important factors for separating L-Lb21 from L-OP50 (Figure 3C). These include metabolites involved in energy metabolism (isocitrate, succinate, AMP, carnitine and galactitol), and in osmoregulation (carnitine and betaine). The level of 4-aminobutyrate (4-AB), also known as GABA, a metabolite that in bacteria is involved in resistance toward acidic pH, was significantly lower in L-Lb21 compared to L-OP50 (Table 1).

Interestingly, some of the remaining metabolites that are not AAs but identified as important factors discriminating worms fed Lb21 compared to OP50 are also involved in oxidative stress and energy metabolism (Figure 3D). These include higher levels of betaine and malonate, a competitive inhibitor of succinate dehydrogenase of the TCA cycle (Gupta, 2019). In contrast, the levels of glucose and AMP were significantly lower (Table 2). Interestingly, betaine was one of the metabolites with higher levels in L-Lb21 compared to those in L-OP50 (Tables 1, 2).

## Discussion

Probiotic supplementation has been reported by many researchers to prevent and alleviate a wide range of diseases; however, there are also studies reporting no benefit or even negative effects (Suez et al., 2019). The beneficial host-microbiome interaction is complex, as it depends on both the bacterial strains and host genetics. Furthermore, manufacturing and administration protocols can influence the efficacy (Suez et al., 2019; Schifano et al., 2020). In addition, even closely related bacterial strains of the same species may exhibit completely different effects even in the same host (Mørch et al., 2021). Thus, one of the main limitations for fully utilizing probiotics as alternatives to traditional antibiotics is the lack of knowledge about the underlying mechanisms, not least at the metabolite level of both the bacteria and the host.

Well-characterized genetic model organisms, like *C. elegans*, are highly suited for metabolomic studies. In this study, we have performed the mechanistic analysis of the Lb21 probiotic response one step further and investigated changes at the metabolite level due to a probiotic diet.

### You are not simply what you eat

Using NMR-based metabolomics, we could identify the top 20 most important metabolites in terms of differentiating probiotics and regular OP50 food as well as the corresponding host metabolomes.

**Figure 4:**
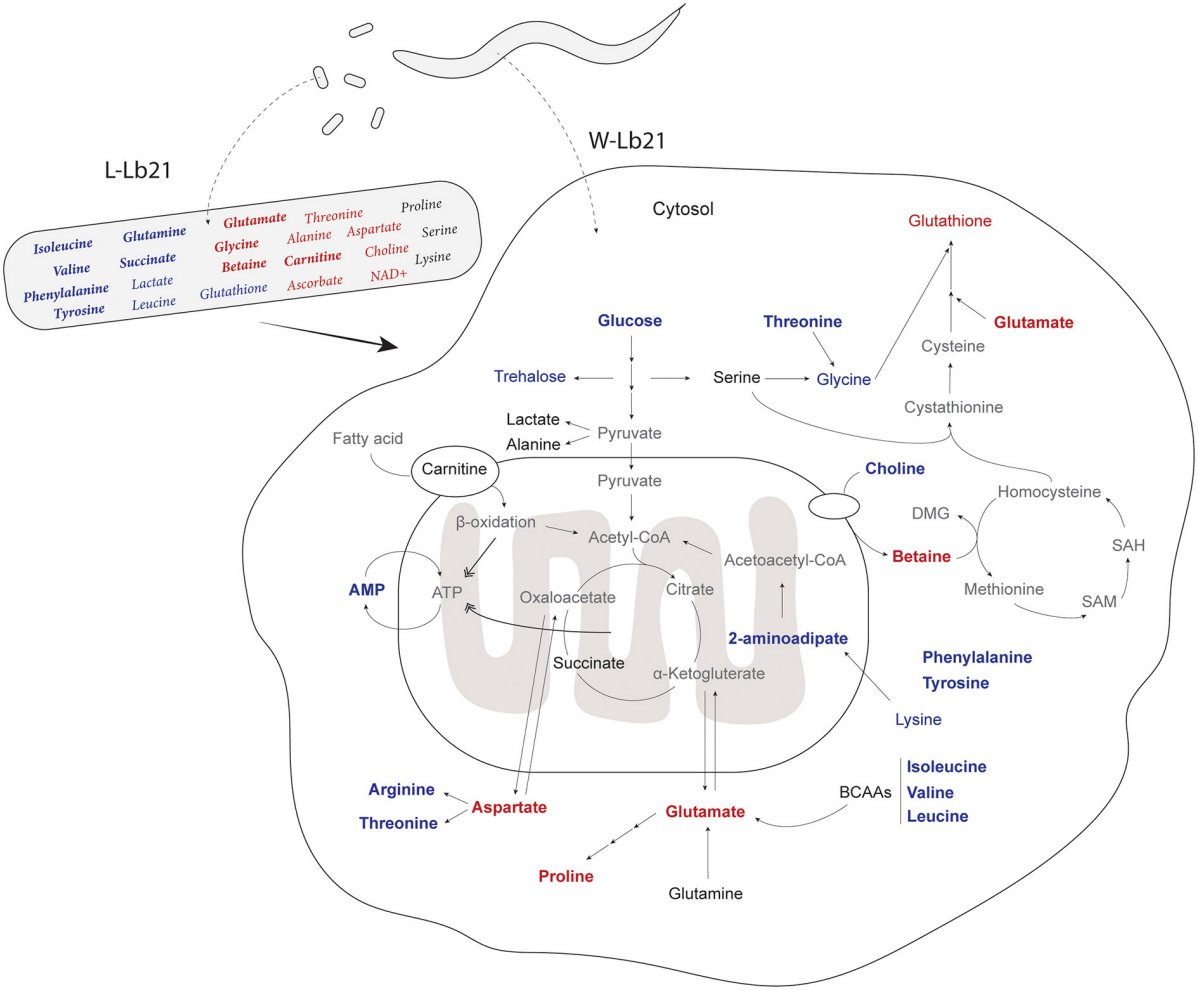
Metabolic characterization of *C. elegans* fed Lb21: Metabolites that are significantly differently identified by *t*-test between L-OP50 and L-Lb21 or W-OP50 and W-LB21 are colored; red are increased, blue are decreased, and those without significant changes in black. Metabolites in bold are one of the 20 metabolites identified by the sPLS-DA analysis. Metabolites in gray were not assigned.

We found that many of the same metabolites identified as important factors in Lb21 bacteria compared to OP50 are also the same identified in the worms fed the two different bacterial diets (Figure 4). When changing the diet of the worms, we expect to find alterations in the metabolome of the host, and some of these will be due to a different nutritional metabolome composition of the new food and other changes will result from alterations of the host metabolism. Indeed, not all metabolites show a proportional relationship between diet and host. For example, the levels of threonine, choline, and glycine were higher in L-Lb21 compared to those in L-OP50, but lower in worms fed Lb21 compared to worms fed OP50 (Tables 1, 2). The opposite was seen for glutathione which had lower levels in Lb21 bacteria compared to OP50 but higher levels in worms fed Lb21 compared to worms fed OP50 (Tables 1, 2). Furthermore, proline and arginine were not significantly different between L-Lb21 and L-OP50, but worms eating Lb21 had significantly higher levels of proline and lower levels of arginine compared to worms eating OP50 (Table 2). The level of the coenzyme nicotinamide adenine dinucleotide (NAD+) was significantly higher in L-Lb21 compared to L-OP50 but not detected in the worms (Table 1). Glutamine is an example of an opposite trend with lower levels in L-Lb21 compared to L-OP50 but similar levels in the worms fed both. These data emphasize the importance of profiling both the bacterial food source and the host response when trying to correlate metabolic changes with specific phenotypes and ultimately identify causal metabolites.

### The probiotic effect of Lb21 could be *via* dietary restriction

Our analysis identified 12 AAs with a high impact on the sPLS-DA analysis for both bacteria and nematode samples. Lactic acid bacteria are auxotrophic for many AAs (Deguchi and Morishita, 1992) and require growth media containing these samples. Despite growing Lb21 in MRS media that support the growth of lactic acid bacteria, and being spotted onto NGM plates containing peptone, also a source of AAs, Lb21 was found to have low levels of both essential (isoleucine, phenylalanine, and valine) and non-essential (tyrosine and glutamine) AAs. Interestingly, the levels of non-essential AAs, i.e., glycine and glutamate, and essential threonine were higher compared to those of OP50. This indicates that AA metabolism is considerably altered in the Lb21 compared to OP50, which in turn likely affects the host.

Like humans, *C. elegans* cannot synthesize all AAs *de novo* and thus needs to obtain essential AAs from the diet (Zeči et al., 2019). We noted that the levels of all the identified essential AAs were significantly lower in worms fed Lb21 compared to worms fed the standard OP50 food, including threonine despite its higher level in the Lb21 lawn compared to OP50. Depletion of AAs could cause dietary restriction (DR), which is the reduction of food consumption below *ad libitum* without causing malnutrition. DR is known to increase the life span and stress resistance in many different organisms (Kapahi et al., 2017; Green et al., 2021). Interestingly, iso-caloric modulation of specific AAs affects health and life span. In the fruit fly *Drosophila melanogaster*, a longevity-promoting DR diet supplemented with only the essential AAs restores the life span to the level observed for normal feeding. Thus, a lack of essential AAs was concluded to be the explanation for life span extension of DR (Grandison et al., 2009). If the underlying molecular mechanisms are conserved, a Lb21-diet could perhaps confer a similar DR effect. However, in *C. elegans*, individual supplementation of AAs to an *E. coli* HT115-diet also increases life span, except for phenylalanine and aspartate (Edwards et al., 2015). Both increased and decreased levels of AAs can cause life span extension that demonstrates the complex interplay between AAs and DR-related mechanisms.

There are several DR paradigms established in *C. elegans* including bacterial dilution-mediated dietary restriction and knock down of *eat-2*, a nicotinic acetylcholine receptor subunit (Zhang and Mair, 2017). The *eat-2* mutants have prolonged life span due to DR caused by defective pharyngeal function (Lakowski and Hekimi, 1998). Furthermore, in *eat-2* mutants, the level of the isovaleryl-CoA dehydrogenase PAH-1, a key enzyme in AA metabolism, is reduced (Yuan et al., 2012). Interestingly, we have previously reported lower levels of PAH-1 in worms fed Lb21 compared to OP50 (Mørch et al., 2021). This is in line with AA depletion and DR being part of the Lb21 probiotic response.

In a prior study, the metabolomes of seven day-old *eat-2* mutants were analyzed using 1H high-resolution magic-angle spinning nuclear magnetic resonance analysis of intact worms (Pontoizeau et al., 2014). Compared to wildtype worms, the *eat-2* mutants have increased levels of glutamate, glutamine, lysine, succinate, cystathionine, and arginine. Furthermore, they have decreased levels of glycerophosphocholine, formate, leucine, phosphocholine, and trehalose (Pontoizeau et al., 2014). We observed a similar change for glutamate, leucine, and trehalose but the opposite for lysine and arginine when worms are fed a probiotic Lb21 diet. For the remaining metabolites determined in the *eat-2* study, the levels in our experiment are not significantly different or they have not been identified in our NMR analysis. Thus, the metabolomes of worms fed a Lb21 probiotic diet do not directly reflect those of *eat-2* mutants. However, it should be kept in mind that the protocols differ in the NMR instruments used, the use of 5'-fluorodeoxyuridine, and the age of the animals. Age, in particular, has been shown to have a significant influence on the levels of individual AAs (Gao et al., 2017; Liu et al., 2019). Thus, based on the different metabolic profiles, we cannot rule out a Lb21 probiotic mechanism that includes DR.

*C. elegans* increases the expression of antioxidant genes such as *gst-4* and *gst-10* in response to being starved for 1 day (Tao et al., 2017). Our previously published proteome analysis shows that the protein levels of GST-4 and GST-10 are downregulated in worms fed Lb21 compared to worms fed OP50 (Mørch et al., 2021). This indicates that worms fed Lb21 are not starved, consistent with their better health. In fact, it has been suggested that DR is a low intensity stressor and that the beneficial effect is mediated *via* hormesis (Masoro, 1998). This is supported by the fact that glucose restriction increases life span *via* mitohormesis (Schulz et al., 2007). The latter is particularly interesting because we found that worms fed a probiotic Lb21 diet have reduced the levels of glucose. Thus, it is possible that the Lb21 diet protects against MRSA by inducing a beneficial mild stress.

### Lb21-induced metabolic changes do not resemble those of reduced insulin signaling

There are well-documented links between increased stress resistance and longevity and most long-lived mutants are resistant to different types of stress. For example, long-lived mutants in the insulin/IGF-1 signaling pathway are resistant to heat (Lithgow et al., 1994), oxidative stress (Honda et al., 2008), heavy metals (Barsyte et al., 2001), protein misfolding (Morley et al., 2002; Cohen et al., 2006), and pathogen infection (Garsin et al., 2003; Evans et al., 2008).

The metabolomes of different long-lived mutants (several *daf-2* alleles, *daf-28*, and *ife-2*) are distinctly different from wild type but rather similar to each other (Fuchs et al., 2010). Common longevity signature metabolites include increased levels of trehalose, branched-chain amino acids (BCAAs, leucine, isoleucine, and valine) and glutamine, and decreased levels of choline, betaine, and glutamate (Fuchs et al., 2010). Many of these metabolomic changes have been confirmed by others (Martin et al., 2011; Castro et al., 2013; Davies et al., 2015). Interestingly, in another study, a *sir-2.1* mutant with a slightly shorter life span exhibited the exact opposite change of these metabolites (An et al., 2012). Furthermore, supplementation with trehalose (Honda et al., 2010; Seo et al., 2018) and BCAAs (Mansfeld et al., 2015) increases life span. The mechanism of Lb21-increased life span must be different as we found that Lb21-fed worms show significantly decreased levels of trehalose and BCAAs, and significantly increased levels of betaine and glutamate. The metabolic changes due to a Lb21 diet do not align with those of reduced insulin signaling mutants which is consistent with our observation that MRSA protection by Lb21 is not dependent on the FOXO transcription factor DAF-16 (Mørch et al., 2021).

### Putative probiotic metabolites

Prior to this study, others have used *C. elegans* as a screening platform to identify new probiotic bacterial strains (Christensen et al., 2017). However, only a few studies have addressed the metabolome of probiotic bacteria and/or worms feeding on them. A study of three *Lactobacillus delbrueckii* subspecies found them to affect the *C. elegans* life span in opposite directions and only one increased the longevity (Zanni et al., 2017). The metabolomic analysis revealed that the life span extending *L. delbrueckii* subsp. *bulgaricus*, a commercially available strain, contained higher levels of glutamate, aspartate, glycine, asparagine, and serine. Interestingly, Lb21 also has significantly more glutamate, aspartate, and glycine compared to OP50. This suggests that these metabolites could be important for the Lb21 probiotic effect. Previous studies support this result, as glutamate, glycine, and proline supplementation extends life span in worms (Edwards et al., 2015; Liu et al., 2019). As glutamate can be converted to proline, the increased level of proline in Lb21-fed animals is consistent with a high level of glutamate. Glutamate supplementation also increases the life span of yeast (Wu et al., 2013).

Furthermore, glutamate plays a central role in metabolism as it acts as the donor of amino groups for many nitrogen-containing metabolites, and it can feed into the TCA cycle to provide energy (Walker and van der Donk, 2016). Glutamate is also used in the *de novo* synthesis of the antioxidant glutathione, a tripeptide made from glutamate, cysteine, and glycine (Ferguson and Bridge, 2019). The significantly higher levels of glutamate and glycine in L-Lb21 compared to L-OP50 support the synthesis of glutathione, a tripeptide made from glutamate, cysteine, and glycine (Ferguson and Bridge, 2019). In line with this, glutathione was significantly increased in worms fed Lb21. Interestingly, *C. elegans* fed a human fecal microbiota transplantation sample promotes the production of intracellular glutathione, which in turn improves intestinal barrier function and protects against nano-plastics induced toxicity (Chu et al., 2021). Ascorbate was present in a higher level in the Lb21 bacteria compared to OP50. In living organisms, glutathione and ascorbate act as antioxidants and can protect against reactive oxygen species (ROS). Ascorbate also acts as a cofactor in enzymatic reactions (Padayatty et al., 2003). In terms of novel MRSA treatment strategies, antioxidants are receiving increased attention because biofilm development might involve ROS signaling (Cattò et al., 2021). Glutathione is particularly interesting with regards to the Lb21 induced MRSA resistance in *C. elegans*, as it inhibits MRSA growth and biofilm architecture *in vitro* (Das et al., 2019). Thus, glutathione could play a role in the increased resistance toward MRSA observed for Lb21-fed worms.

The levels of glycine are about 10-fold higher in Lb21 bacteria compared to OP50. This is interesting, because glycine supplementation increases the longevity *via* the methionine cycle where it appears to be degraded immediately as it does not accumulate in the animals (Liu et al., 2019). Consistent with this, we found that Lb21-fed worms have slightly reduced levels of glycine despite higher concentrations in the Lb21 diet compared to OP50.

The significant increase in aspartate found in both Lb21 and worms fed Lb21 is intriguing as the restriction of aspartate extends the life span of *C. elegans* and yeast (Wu et al., 2013; Edwards et al., 2015). However, as the levels of several AAs are different in L-Lb21 compared to L-OP50, a higher level of aspartate under these circumstances could be beneficial for the host.

Feeding *C. elegans* with a Lb21 diet increases life span (Mørch et al., 2021). Therefore, the increased level of NAD+ in L-Lb21 compared to L-OP50 is interesting because NAD+ supplementation has been shown to have anti-aging effects and delay the onset of age-related diseases (Fang et al., 2017).

The increased level of betaine found in worms fed Lb21 could play a role in the protective effect against stress as supplementation with betaine reduces protein aggregation induced paralysis in an Alzheimer's disease *C. elegans* model (Leiteritz et al., 2018). The paralysis-reducing effect of betaine was found to be dependent on the enzyme cystathionin-β-synthase (*cbs-1*). Whether *cbs-1* is a target for betaine in relation to the MRSA-resistant effect of Lb21 remains to be elucidated.

In summary, many of the important metabolites identified in this study, either in the probiotic bacteria Lb21 or in the host following a probiotic diet, have previously been described as health promoting ones. It remains to be shown if there is a single causal metabolite or if MRSA resistance results from the combined effect of multiple metabolites.

### Metabolites from probiotic bacteria as novel antibiotic alternatives

Given the global rise in multidrug resistant pathogenic bacteria, alternatives to traditional antibiotics are urgently needed. There is increasing evidence from a range of organisms and model systems that some probiotic bacteria can be as effective as traditional antibiotics. However, using live bacteria for the prevention or treatment of infections is not trivial and requires stringent production methods, storage conditions, and treatment protocols. We have previously shown that the genetic background of the host affects whether the given probiotic bacteria will have a beneficial effect (Mørch et al., 2021). This could be a concern for the shelf products, but this suggests that there is great potential for personalized precision medicine. Many of the inherent limitations of using live organisms could be overcome if the beneficial metabolites could be identified and isolated. This requires a comprehensive characterization of both the bacterial metabolomes and host responses to these across many different probiotic strains and hosts. In this study, we provided a set of metabolites that potentially could lead to strategies for protection against MRSA, as the Lb21 probiotics offer very strong MRSA resistance in *C. elegans*.

## Data availability statement

The raw data supporting the conclusions of this article will be made available by the authors, without undue reservation.

## Author contributions

AO, FM, and KM contributed to conception and design of the study. KM, MM, and MH performed the experiments. HN and KM analyzed the NMR data. AO, HN, and KM wrote the manuscript. All authors contributed to manuscript revision and approved the submitted version.

## Funding

Funding was provided by Innovation Foundation Denmark grant number 4105-00019B. The *C. elegans* NL2099 *rrf-3 (pk1426)* strain was purchased from CGC, which is funded by NIH Office of Research Infrastructure Programs. The authors would like to thank Dr. Arthur Ouwehand (Global Health & Nutrition Sciences, International Flavors and Fragrances, Kantvik, Finland) for providing the Lb21 strain. The community-acquired isolate MRSA 43484 was provided by Statens Serum Institut, Copenhagen, Denmark. Finally, access to the NMR spectrometers at the Danish Center for Ultrahigh-Field NMR Spectroscopy (Ministry of Higher Education and Science grant AU- 2010-612-181) is gratefully acknowledged.

## Conflict of interest

The authors declare that the research was conducted in the absence of any commercial or financial relationships that could be construed as a potential conflict of interest.

## Publisher's note

All claims expressed in this article are solely those of the authors and do not necessarily represent those of their affiliated organizations, or those of the publisher, the editors and the reviewers. Any product that may be evaluated in this article, or claim that may be made by its manufacturer, is not guaranteed or endorsed by the publisher.
